# Postulated Role of Vasoactive Neuropeptide-Related Immunopathology of the Blood Brain Barrier and Virchow-Robin Spaces in the Aetiology of Neurological-Related Conditions

**DOI:** 10.1155/2008/792428

**Published:** 2009-02-16

**Authors:** D. R. Staines, E. W. Brenu, S. Marshall-Gradisnik

**Affiliations:** ^1^Gold Coast Population Health Unit, Queensland Health, Southport, Gold Coast, Queensland 4215, Australia; ^2^Population Health and Neuroimmunology Unit, Faculty of Health Science and Medicine, Bond University, Robina, Queensland 4229, Australia

## Abstract

Vasoactive neuropeptides (VNs) such as pituitary adenylate cyclase-activating polypeptide (PACAP) and vasoactive intestinal peptide (VIP) have critical roles as neurotransmitters, vasodilators including perfusion and hypoxia regulators, as well as immune and nociception modulators. They have key roles in blood vessels in the central nervous system (CNS) including maintaining functional integrity of the blood brain barrier (BBB) and blood spinal barrier (BSB). VNs are potent activators of adenylate cyclase and thus also have a key role in cyclic AMP production affecting regulatory T cell and other immune functions. Virchow-Robin spaces (VRSs) are perivascular compartments surrounding small vessels within the CNS and contain VNs. Autoimmunity of VNs or VN receptors may affect BBB and VRS function and, therefore, may contribute to the aetiology of neurological-related conditions including multiple sclerosis, Parkinson's disease, and amyotrophic lateral sclerosis. VN autoimmunity will likely affect CNS and immunological homeostasis. Various pharmacological and immunological treatments including phosphodiesterase inhibitors and plasmapheresis may be indicated.

## 1. INTRODUCTION

Vasoactive neuropeptides (VNs) (e.g., pituitary adenylate cyclase-activating polypeptide (PACAP) and vasoactive intestinal peptide (VIP)) are widely distributed in the central nervous system (CNS) and peripheral nervous system (PNS) including autonomic nervous system (ANS) and peripheral tissues including heart, lung, pancreas, adrenal gland, gonads, and gastrointestinal tract as well as immune cells and lymphatic system [1].

VNs have critical roles and functions as neurotransmitters and neuroregulators, neurotrophic stimulators, hormonal regulators, vasodilators (including perfusion and hypoxia regulators), as well as immune and nociception modulators. They have key roles in blood vessel function in the CNS and contribute to high-level neurological functions including memory and learning [2]. They have a well-described neuroprotective role [3] and act as inflammatory mediators in microglial activation [4].

VNs exert potent effects in metabolism as they have a vital role in cyclic adenosine monophosphate (cAMP) production and regulation through adenylate cyclase (AC) activation. Immunological dysregulation of vital biochemical and/or epigenetic mechanisms affecting VNs resulting in down-regulation of cAMP are possible pathways by which disease entities become manifest. Their role along with other neurotrophic factors [5] in maintaining cAMP levels [6] may be of vital importance for the integrity of blood brain barrier (BBB) and blood spinal barrier (BSB).

BBB and BSB have traditionally been regarded as the main barriers between the brain and spinal cord parenchyma and the intravascular compartment and are, therefore, important in keeping immune cells and macromolecules from interfering with brain and spinal neurological processes. However, disruption of BBB and BSB is well known in certain pathological states suggesting that they function more as a “sieve” rather than an absolute barrier.

Virchow-Robin spaces (VRSs) are compartments surrounding small vessels within the CNS which contain interstitial fluid and, while some functional contact with subarachnoid spaces may occur for solute exchange, they may not contain CSF as commonly thought [7]. VRSs have important connections with lymphatic drainage of the head and neck [8], having intricate pial relations and providing a surface for activity of neuropeptides, hormones, and cytokines. Pial cells may have a role in protecting the brain from exogenous catecholamines [9], and VRS may have a complex role in leukocyte recruitment across the BBB [10].

VNs are known to have neuroprotective effects through hypoxia protection on passage through the BBB [11] via a transport mechanism which enables the intact peptides to enter the parenchymal space of the brain [12]. Additionally, VNs have protective effects on neurons and glial cells [13]. VNs may have a significant role in blood BBB/BSB function and likely assist in immune regulation of VRS in the brain and spinal cord. The present paper asserts that in view of the many vital roles of VNs in CNS neuroregulatory and immunological function including BBB function, autoimmunity to VNs or VN receptors will have significant effects on homeostasis resulting in disease states.

## 2. VASOACTIVE NEUROPEPTIDES IN IMMUNOLOGICAL CONTROL

VNs exert influence over inflammatory control mechanisms including influencing Th1 to Th2 shift, and the suppression of TNF alpha [14] has been established in cAMP participation in vascular dysfunction involving endothelial cells [15]. The VRS has been identified as a location for immunoreactive lymphocytes in penetration of neuronal parenchyma [16]. Also, VIP has been identified in connection with neuronal function and VRS suggesting that VIP may have a regulatory function associated with vasodilatation [17]. We assert that important immunoregulation occurs in VRS and that this may involve VN regulation. Many regulatory functions are dependent on IL-10 and IL-4, these may be compromised in VN failure. Moreover, leakiness in BBB and BSB functions may encourage development or relapses in neurological conditions, such as multiple sclerosis (MS) [18].

Regulatory T cells (Tregs) function to control autoreactive T cells in the periphery [19]. Moreover, Treg function is substantially influenced by VNs [20] and may also have influence over Th17 direction [21]. Loss of Treg function in VRS will, therefore, have significant implications for inflammatory control. Moreover, Th17 development occurs under IL-6 and TGF beta influence [22], and this may be a key switching point from a protective Treg to an autoimmune Th17 phenotype. Certainly, Th1 and Th17 ratios are important in brain and spinal inflammation regulation [23].

Interestingly, a number of seemingly unrelated disorders may be implicated in this postulated pathology. For example, Crohn's disease (CD) and MS are inflammatory disorders with known Th1-directed cytokines and loss of Foxp3 Treg function [24, 25].

As VRS have important roles in controlling macrophage and perivascular infiltrates in MS [26], their immunological function in relation to VNs becomes of considerable interest. Complement-fixing myelinolytic antigens have been identified in the VRS in early MS [27], indicating the possible involvement of VRS in immune activity.

## 3. POSTULATED VASOACTIVE NEUROPEPTIDE AUTOIMMUNITY AND THE BLOOD BRAIN BARRIER

VN autoimmunity has been postulated as a contributing cause for some fatigue-related conditions [28]. The present paper explores the possible role VN immune dysregulation may have on VRS function and CNS activity as this anatomical pathway may have a significant bearing on disease aetiology.

Because VNs are widely distributed in the CNS, neurovascular, and immune systems, they exert extraordinary influence on neurological, vascular, and immunological functions. Significantly, VNs exert mostly anti-inflammatory activities and loss of their function, for example, through autoimmune compromise, could become manifest as unmodulated activation of immune responses. Autoimmunity directed at VN guanine protein-coupled receptors (GPCRs) is currently unproven and loss-of-function autoimmunity to GPCRs generally is not widely documented. However, parallels exist with other conditions such as Sjogren's syndrome which has T cell and/or B cell antibody targeting of acetylcholine GPCRs [29]. The role of VNs in linking the innate and acquired immune systems [30] suggests that there would be significant effects on homeostasis if compromised.

Pain, fatigue, and dysregulation of nociception may be prominent features in these syndromes as a result of VN compromise in the CNS. Pain and nociception are mediated through spinal and cerebral pathways particularly via spinothalamic, spinoreticular, and spinomesencephalic structures. The periaqueductal grey (PAG) region surrounding the third ventricle and cerebral aqueduct is a key area for regulation of noxious stimuli. This area has high-density VN presence and may be a prominent target for VN autoimmune compromise. Similarly, VRS have a critical role in maintaining the interstitial fluid immunological milieu and may be a vulnerable area for VN compromise.

It is likely that BBB, BSB, and VRS play an important role in immunological maintenance and homeostasis within the CNS. Macrophages in VRS express MHC class II antigens and interact with lymphocytes from the blood in initiating and promoting immune responses to foreign antigens in the brain. Immunological activity within VRS may have a role in a number of neurological conditions. Moreover, sites deficient in BBB include the subarachnoid space and pial surface, and circumventricular organs may be more prone to macromolecule penetration of CSF, and this may have implications regarding autoimmune dysfunction within the CNS [31]. Collections of macrophages may occur in VRS following trauma in a proinflammatory context [32] and pathological dilatation of VRS may occur from a variety of causes including ischaemia [33].

VRS distribution in anatomically specific locations reflects the functional attributes of these locations. For example, VRS in the nucleus tractus solitarius in the dorsal medulla oblongata suggests they may have a role in viscerosensory and autonomic functions [34]. Capillary diversity within the subfornical organ (SFO) and area postrema (AP) may function as low-resistance pathways for the rapid dispersion of blood-borne hormones inside their organ boundaries and this may have a role in the regulation of blood pressure and body fluids [35]. Hence, these functions linked to VRS microanatomy may be particularly susceptible to VN compromise.

## 4. POSTULATED CEREBROVASCULAR AND CNS EFFECTS OF VN FAILURE

Vascular compromise within the CNS possibly as a result of VN compromise may give rise to features consistent with certain forms of dementia. Fronto-temporal dementia (FTD) is a neurodegenerative disease in which a vascular component is suggested and immunoreactivity of Bax, a proapoptotic protein regulated in part by VNs in astrocytes, suggests a role for autoimmunity in the pathology of FTD [36]. Astrogliosis in FTD corresponds with SPECT hypoperfusion, suggesting that astrocyte disruption may be related to disturbances of cerebral perfusion in FTD [37]. Cognitive dysfunction is associated with reduced cerebral blood flow in different types of dementia [38]. Moreover, VRS dilatation associated with microvessel abnormality may contribute to the diagnosis of vascular dementias [39]. Changes in social behaviour occur in cerebrovascular comprise and may result from an FTD-like syndrome [40]. Similarly, reduction in cortical blood flow has been identified in CFS patients [41, 42]; however, these findings were not replicated in a study of twins with CFS [43].

Astroglial water channel aquaporin (AQP4) is essential for the maintenance of blood-brain barrier integrity [44]. Antibodies to AQP4 have a highly specific role in neuromyelitis optica (NMO) and characteristically bind to cerebral microvessels, pia mater, and VRSs [45]. Secretin is important for other aquaporin expression via vasopressin. Secretin receptor-null mice, for example, have reduced renal expression of AQP2 and AQP4 [46]. Interestingly, VIP, which is related to secretin, also has a relationship with aquaporin distribution and function. An association with VRS has been identified supporting the view that cortical nerve cells release VIP in the perivascular space during periods of activity and thus contribute to local vasodilatation associated with neuronal function. There is an important relationship whereby ATP over expression has a down-regulatory impact on AQP4 expression [47]. Adenylate cyclase compromise could have an impact on ATP levels by failure to convert to cAMP, arguably maintaining high levels of ATP with adverse consequences for AQP4 function. Thus, VN failure may present with dementia-like signs and symptoms.

## 5. CONCLUSIONS

Neurological conditions often present with fatigue and other symptoms including memory and concentration loss, emotional lability, and confusion. Multisystem involvement including cerebrospinal effects of these conditions may be explained in part through VN compromise. In particular, cerebrovascular and spinovascular compromise acting at the ultra-microscopic level involving BBB and BSB may contribute to these disorders. Compromise of VN receptors critical to BBB and BSB functioning may have a role in these disorders and should be the subject of further research.

This paper asserts that CNS vascular compromise including immunological dysfunction of the VRS may be linked to postulated VN autoimmunity. Evidence for compromise of VN receptors at different levels including VN receptor mRNA, protein transcription, cellular migration and trafficking, cell membrane localisation, as well as possible antibody or T cell targeting will be required to support this hypothesis.

There are significant treatment implications from this hypothesis. VNs, such as PACAP and VIP, exert potent effects in metabolism as they have a vital role in cAMP production and regulation through AC activation. Phosphodiesterase (PDE) enzymes metabolise cAMP as a means of feedback regulation of cAMP levels. VN compromise will result in impaired AC activation and hence impaired cAMP production. Thus, phosphodiesterase inhibitors (PDEIs), novel therapeutic substances used to promote cAMP levels, may have a role in treatment of VN autoimmune conditions.

Proof of this hypothesis will require demonstration of pathological antibodies or T cells specific to VN immunological pathology. Alternatively, antagonistic variants of VNs themselves may exist.
